# (Acetato-κ*O*)(acetato-κ^2^ *O*,*O*′)[2-(3,5-di­methyl-1*H*-pyrazol-1-yl-κ*N* ^2^)quinoline-κ*N*]zinc(II)

**DOI:** 10.1107/S1600536812025664

**Published:** 2012-06-13

**Authors:** Muhd. Hidayat bin Najib, Ai Ling Tan, David J. Young, Seik Weng Ng, Edward R. T. Tiekink

**Affiliations:** a Faculty of Science, Universiti Brunei Darussalam, Jalan Tungku Link BE 1410, Negara Brunei Darussalam; b Department of Chemistry, University of Malaya, 50603 Kuala Lumpur, Malaysia; c Chemistry Department, Faculty of Science, King Abdulaziz University, PO Box 80203 Jeddah, Saudi Arabia

## Abstract

The Zn^II^ atom in the title compound, [Zn(C_2_H_3_O_2_)_2_(C_14_H_13_N_3_)], is coordinated by an N_2_O_3_ donor set defined by the quinolinyl- and pyrazolyl-N atoms of the chelating heterocyclic ligand, and three carboxyl­ate-O atoms derived from the monodentate and bidentate carboxyl­ate ligands. Distortions from the ideal square-pyramidal coordination geometry relate to the restricted bite angle of the chelating ligands, *i.e*. O—Zn—O = 59.65 (5) and N—Zn—N = 76.50 (6)°, and the close approach of the non-coordinating carbonyl atom [Zn⋯O = 2.858 (2) Å]. In the crystal, mol­ecules are consolidated into a three-dimensional architecture by C—H⋯O inter­actions

## Related literature

For background to luminescent coordination complexes, see: Bai *et al.* (2011 ▶, 2012 ▶); Chou *et al.* (2011 ▶); Wang (2001 ▶). For the synthesis, see: Savel’eva *et al.* (2009 ▶); Scott *et al.* (1952 ▶). For the structure of the dichlorido analogue, see: Najib *et al.* (2012 ▶). For additional geometric analysis, see: Addison *et al.* (1984 ▶).
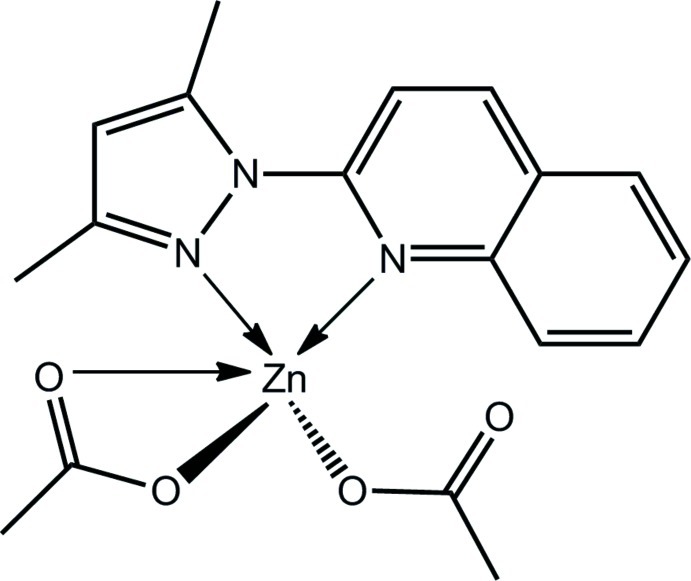


## Experimental

### 

#### Crystal data

[Zn(C_2_H_3_O_2_)_2_(C_14_H_13_N_3_)]
*M* *_r_* = 406.73
Triclinic, 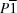

*a* = 7.6586 (4) Å
*b* = 10.7334 (6) Å
*c* = 11.5772 (4) Å
α = 69.437 (4)°
β = 81.546 (3)°
γ = 72.736 (4)°
*V* = 849.93 (7) Å^3^
*Z* = 2
Cu *K*α radiation
μ = 2.27 mm^−1^
*T* = 100 K
0.25 × 0.15 × 0.05 mm

#### Data collection

Agilent SuperNova Dual diffractometer with Atlas detector
Absorption correction: multi-scan (*CrysAlis PRO*; Agilent, 2012 ▶) *T*_min_ = 0.617, *T*_max_ = 1.000
6205 measured reflections
3498 independent reflections
3322 reflections with *I* > 2σ(*I*)
*R*_int_ = 0.021

#### Refinement

*R*[*F* ^2^ > 2σ(*F* ^2^)] = 0.030
*wR*(*F* ^2^) = 0.081
*S* = 1.03
3498 reflections
239 parameters
H-atom parameters constrained
Δρ_max_ = 0.67 e Å^−3^
Δρ_min_ = −0.45 e Å^−3^

### 

Data collection: *CrysAlis PRO* (Agilent, 2012 ▶); cell refinement: *CrysAlis PRO*; data reduction: *CrysAlis PRO*; program(s) used to solve structure: *SHELXS97* (Sheldrick, 2008 ▶); program(s) used to refine structure: *SHELXL97* (Sheldrick, 2008 ▶); molecular graphics: *ORTEP-3* (Farrugia, 1997 ▶) and *DIAMOND* (Brandenburg, 2006 ▶); software used to prepare material for publication: *publCIF* (Westrip, 2010 ▶).

## Supplementary Material

Crystal structure: contains datablock(s) global, I. DOI: 10.1107/S1600536812025664/hb6839sup1.cif

Structure factors: contains datablock(s) I. DOI: 10.1107/S1600536812025664/hb6839Isup2.hkl

Additional supplementary materials:  crystallographic information; 3D view; checkCIF report

## Figures and Tables

**Table 1:** Selected bond lengths (Å)

Zn—O1	2.0388 (14)
Zn—O2	2.3240 (15)
Zn—O3	1.9397 (13)
Zn—N1	2.0570 (15)
Zn—N3	2.1460 (14)

**Table 2:** Hydrogen-bond geometry (Å, °)

*D*—H⋯*A*	*D*—H	H⋯*A*	*D*⋯*A*	*D*—H⋯*A*
C4—H4*B*⋯O3^i^	0.98	2.57	3.544 (2)	176
C5—H5*A*⋯O2^ii^	0.98	2.60	3.417 (3)	141
C7—H7⋯O2^ii^	0.95	2.56	3.235 (2)	128
C9—H9*C*⋯O4^iii^	0.98	2.36	3.274 (2)	156
C12—H12⋯O1^iv^	0.95	2.51	3.310 (2)	142

Symmetry codes: (i) 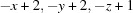; (ii) 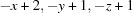; (iii) 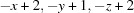; (iv) 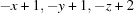.

**Fig. 1.**
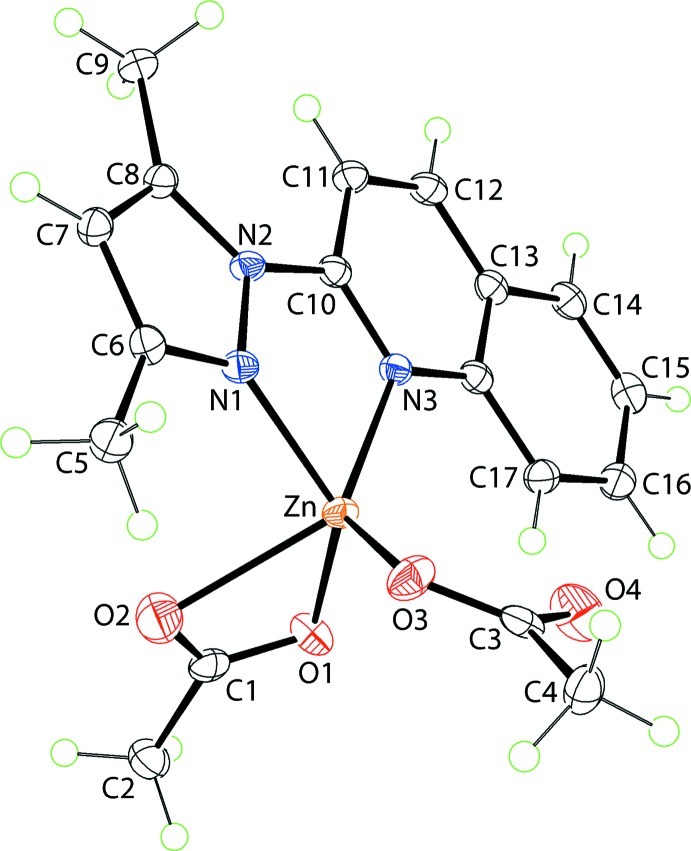
The molecular structure of (I) showing displacement ellipsoids at the 50% probability level.

**Fig. 2.**
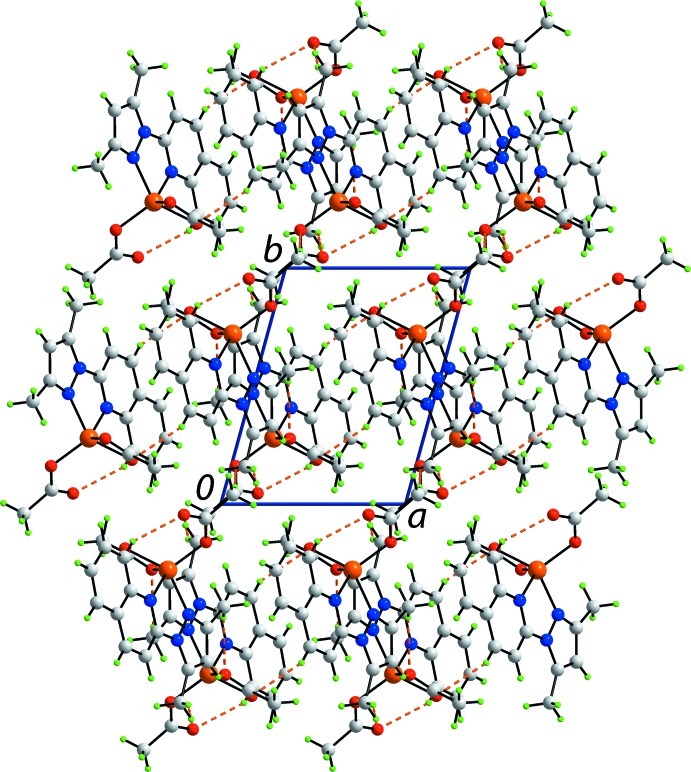
A view of the unit-cell contents of (I) in projection down the c axis. The C—H···O interactions are shown as orange dashed lines.

## supplementary crystallographic information

### Comment

Many Zn^II^ complexes of nitrogen-containing ligands exhibit intense emission at room temperature (Wang, 2001; Chou *et al.*, 2011; Bai *et al.*, 2011; Bai *et al.*, 2012). The title compound was prepared as part of a series of potentially luminescent coordination complexes for use in organic light emitting diode (OLED) materials. We have previously reported the solid-state structure of dichlorido[2-(3,5-dimethyl-1*H*-pyrazol-1-yl-2)quinoline]zinc(II) (Najib *et al.*, 2012), *i.e*. the dichlorido analogue of the title compound, (I).

The Zn^II^ atom in (I), Fig. 1, is chelated by quinolinyl- and pyrazolyl-N atoms of the heterocyclic ligand, and three carboxylate-O atoms derived from the monodentate and bidentate carboxylates, Table 1. The resulting N_2_O_3_ donor set defines an approximate square pyramid with the Zn atom lying 0.8591 (8) Å out of the plane defined by the O1, O2, N1 and N3 atoms [r.m.s. deviation = 0.1122 Å] in the direction of the O3 atom. The assignment of coordination geometry is quantified by the value of τ = 0.06 which compares to the τ values of 0.0 and 1.0 for ideal square pyramidal and trigonal bipyramidal geometries, respectively (Addison *et al.*, 1984). Significant distortions in the coordination geometry are apparent owing the restricted bite angles of the chelating ligands, *i.e*. O1—Zn—O2 = 59.65 (5)° and N1—Zn—N3 = 76.50 (6)°. Further distortions are related to the relatively close approach of the O4 atom to Zn, the Zn···O4 separation is 2.858 (2) Å. The five-membered chelate ring is approximately planar with a r.m.s. deviation = 0.088 Å and with maximum deviations of 0.074 (2) and -0.057 (1) Å for the N1 and Zn atoms, respectively. The bidentate ligand is planar with the dihedral angle between the quinolinyl and pyrazolyl rings being 2.14 (6)°.

Molecules are consolidated into a three-dimensional architecture by C—H···O interactions, Fig. 2 and Table 2.

### Experimental

The title compound was prepared by modification of a literature procedure (Savel'eva *et al.*, 2009) and as previously described for the corresponding dichloride (Najib *et al.*, 2012). 3,5-Dimethyl-1-(2'-quinolyl)pyrazole (0.0908 g), prepared as in the literature (Scott *et al.*, 1952), in a mixture of EtOH (4 ml) and CH_2_Cl_2_ (2 ml) was added to a suspension of Zn(OAc)_2_ (0.0764 g) in EtOH (8 ml). The solution was heated to dissolve the Zn(OAc)_2_. Light-brown prisms formed over a period of 16 h and were collected by filtration, washed with EtOH and recrystallized from CH_2_Cl_2_/hexane. Yield 0.0733 g (44%). *M*.pt: 474 K. IR v/cm^-1^: 2925, 2864, 2365, 2323, 1604, 1507, 1424, 1388.

### Refinement

Carbon-bound H-atoms were placed in calculated positions [C—H = 0.95–0.98 Å, *U*_iso_(H) = 1.2–1.5*U*_eq_(C)] and were included in the refinement in the riding model approximation.

### Crystal data

**Table:**

[Zn(C_2_H_3_O_2_)_2_(C_14_H_13_N_3_)]	*Z* = 2
*M**_r_* = 406.73	*F*(000) = 420
Triclinic, *P*1	*D*_x_ = 1.589 Mg m^−^^3^
Hall symbol: -P 1	Cu *K*α radiation, λ = 1.54184 Å
*a* = 7.6586 (4) Å	Cell parameters from 3775 reflections
*b* = 10.7334 (6) Å	θ = 4.6–76.3°
*c* = 11.5772 (4) Å	µ = 2.27 mm^−^^1^
α = 69.437 (4)°	*T* = 100 K
β = 81.546 (3)°	Prism, light-brown
γ = 72.736 (4)°	0.25 × 0.15 × 0.05 mm
*V* = 849.93 (7) Å^3^	

### Data collection

**Table:**

Agilent SuperNova Dual diffractometer with Atlas detector	3498 independent reflections
Radiation source: SuperNova (Cu) X-ray Source	3322 reflections with *I* > 2σ(*I*)
Mirror monochromator	*R*_int_ = 0.021
Detector resolution: 10.4041 pixels mm^-1^	θ_max_ = 76.5°, θ_min_ = 4.6°
ω scan	*h* = −9→9
Absorption correction: multi-scan (*CrysAlis PRO*; Agilent, 2012)	*k* = −12→13
*T*_min_ = 0.617, *T*_max_ = 1.000	*l* = −11→14
6205 measured reflections	

### Refinement

**Table:**

Refinement on *F*^2^	Primary atom site location: structure-invariant direct methods
Least-squares matrix: full	Secondary atom site location: difference Fourier map
*R*[*F*^2^ > 2σ(*F*^2^)] = 0.030	Hydrogen site location: inferred from neighbouring sites
*wR*(*F*^2^) = 0.081	H-atom parameters constrained
*S* = 1.03	*w* = 1/[σ^2^(*F*_o_^2^) + (0.0448*P*)^2^ + 0.5376*P*] where *P* = (*F*_o_^2^ + 2*F*_c_^2^)/3
3498 reflections	(Δ/σ)_max_ = 0.001
239 parameters	Δρ_max_ = 0.67 e Å^−^^3^
0 restraints	Δρ_min_ = −0.45 e Å^−^^3^

### Special details

**Table:**

Geometry. All e.s.d.'s (except the e.s.d. in the dihedral angle between two l.s. planes) are estimated using the full covariance matrix. The cell e.s.d.'s are taken into account individually in the estimation of e.s.d.'s in distances, angles and torsion angles; correlations between e.s.d.'s in cell parameters are only used when they are defined by crystal symmetry. An approximate (isotropic) treatment of cell e.s.d.'s is used for estimating e.s.d.'s involving l.s. planes.
Refinement. Refinement of *F*^2^ against ALL reflections. The weighted *R*-factor *wR* and goodness of fit *S* are based on *F*^2^, conventional *R*-factors *R* are based on *F*, with *F* set to zero for negative *F*^2^. The threshold expression of *F*^2^ > σ(*F*^2^) is used only for calculating *R*-factors(gt) *etc*. and is not relevant to the choice of reflections for refinement. *R*-factors based on *F*^2^ are statistically about twice as large as those based on *F*, and *R*- factors based on ALL data will be even larger.

### Fractional atomic coordinates and isotropic or equivalent isotropic displacement parameters (Å^2^)

**Table:**

	*x*	*y*	*z*	*U*_iso_*/*U*_eq_	
Zn	0.81157 (3)	0.72065 (2)	0.729830 (19)	0.01539 (9)	
O1	0.54802 (18)	0.81205 (15)	0.67857 (12)	0.0237 (3)	
O2	0.7235 (2)	0.72283 (17)	0.54526 (14)	0.0333 (3)	
O3	0.97606 (19)	0.83912 (14)	0.66803 (13)	0.0251 (3)	
O4	0.8163 (2)	0.95459 (18)	0.79237 (13)	0.0346 (4)	
N1	0.9903 (2)	0.53183 (15)	0.73964 (13)	0.0157 (3)	
N2	0.9663 (2)	0.42401 (15)	0.84284 (13)	0.0148 (3)	
N3	0.76154 (19)	0.59226 (15)	0.91411 (13)	0.0147 (3)	
C1	0.5715 (3)	0.78835 (18)	0.57607 (17)	0.0191 (3)	
C2	0.4136 (3)	0.8434 (2)	0.49227 (18)	0.0247 (4)	
H2A	0.4333	0.7891	0.4366	0.037*	
H2B	0.4053	0.9401	0.4436	0.037*	
H2C	0.2996	0.8367	0.5421	0.037*	
C3	0.9376 (3)	0.93922 (19)	0.71118 (16)	0.0203 (4)	
C4	1.0519 (3)	1.0407 (2)	0.65732 (18)	0.0238 (4)	
H4A	1.0008	1.1202	0.6865	0.036*	
H4B	1.0515	1.0719	0.5670	0.036*	
H4C	1.1778	0.9961	0.6834	0.036*	
C5	1.1702 (3)	0.56456 (19)	0.54237 (16)	0.0216 (4)	
H5A	1.1823	0.5196	0.4800	0.032*	
H5B	1.2888	0.5773	0.5503	0.032*	
H5C	1.0801	0.6546	0.5171	0.032*	
C6	1.1083 (2)	0.47655 (18)	0.66347 (16)	0.0169 (3)	
C7	1.1602 (2)	0.33201 (18)	0.71639 (16)	0.0172 (3)	
H7	1.2427	0.2686	0.6804	0.021*	
C8	1.0696 (2)	0.29988 (18)	0.82920 (16)	0.0162 (3)	
C9	1.0774 (3)	0.15864 (18)	0.91825 (16)	0.0197 (3)	
H9A	1.1598	0.0897	0.8838	0.029*	
H9B	0.9545	0.1441	0.9330	0.029*	
H9C	1.1231	0.1494	0.9964	0.029*	
C10	0.8445 (2)	0.45914 (18)	0.93770 (15)	0.0146 (3)	
C11	0.8173 (2)	0.35645 (18)	1.04951 (16)	0.0175 (3)	
H11	0.8819	0.2624	1.0634	0.021*	
C12	0.6956 (2)	0.39558 (19)	1.13728 (16)	0.0186 (3)	
H12	0.6735	0.3279	1.2127	0.022*	
C13	0.6026 (2)	0.53597 (18)	1.11662 (16)	0.0165 (3)	
C14	0.4758 (2)	0.5825 (2)	1.20508 (16)	0.0197 (4)	
H14	0.4464	0.5176	1.2803	0.024*	
C15	0.3960 (2)	0.7202 (2)	1.18233 (17)	0.0212 (4)	
H15	0.3129	0.7509	1.2423	0.025*	
C16	0.4366 (2)	0.81699 (19)	1.06979 (17)	0.0204 (4)	
H16	0.3813	0.9126	1.0554	0.025*	
C17	0.5549 (2)	0.77526 (18)	0.98073 (16)	0.0183 (3)	
H17	0.5788	0.8414	0.9047	0.022*	
C18	0.6406 (2)	0.63361 (18)	1.00311 (15)	0.0157 (3)	

### Atomic displacement parameters (Å^2^)

**Table:**

	*U*^11^	*U*^22^	*U*^33^	*U*^12^	*U*^13^	*U*^23^
Zn	0.01744 (13)	0.01236 (13)	0.01501 (13)	−0.00462 (9)	−0.00080 (9)	−0.00227 (9)
O1	0.0225 (7)	0.0315 (7)	0.0164 (6)	−0.0077 (6)	−0.0021 (5)	−0.0058 (5)
O2	0.0279 (8)	0.0360 (8)	0.0346 (8)	0.0037 (6)	−0.0070 (6)	−0.0178 (7)
O3	0.0254 (7)	0.0173 (6)	0.0335 (7)	−0.0087 (5)	−0.0018 (6)	−0.0067 (5)
O4	0.0350 (8)	0.0474 (9)	0.0219 (7)	−0.0177 (7)	0.0070 (6)	−0.0096 (6)
N1	0.0193 (7)	0.0134 (7)	0.0132 (6)	−0.0059 (6)	−0.0005 (5)	−0.0015 (5)
N2	0.0172 (7)	0.0121 (6)	0.0133 (6)	−0.0044 (5)	−0.0012 (5)	−0.0012 (5)
N3	0.0159 (7)	0.0141 (7)	0.0139 (6)	−0.0045 (5)	−0.0019 (5)	−0.0034 (5)
C1	0.0218 (9)	0.0131 (8)	0.0210 (8)	−0.0073 (7)	−0.0013 (7)	−0.0013 (6)
C2	0.0265 (10)	0.0261 (10)	0.0220 (9)	−0.0074 (8)	−0.0060 (7)	−0.0062 (7)
C3	0.0231 (9)	0.0210 (9)	0.0125 (7)	−0.0048 (7)	−0.0062 (6)	0.0009 (6)
C4	0.0294 (10)	0.0212 (9)	0.0253 (9)	−0.0116 (8)	0.0022 (7)	−0.0103 (7)
C5	0.0258 (9)	0.0205 (9)	0.0179 (8)	−0.0085 (7)	0.0030 (7)	−0.0053 (7)
C6	0.0178 (8)	0.0186 (8)	0.0159 (8)	−0.0062 (7)	−0.0010 (6)	−0.0063 (7)
C7	0.0180 (8)	0.0163 (8)	0.0188 (8)	−0.0048 (6)	−0.0019 (6)	−0.0070 (7)
C8	0.0173 (8)	0.0139 (8)	0.0184 (8)	−0.0037 (6)	−0.0040 (6)	−0.0055 (6)
C9	0.0232 (9)	0.0134 (8)	0.0201 (8)	−0.0032 (7)	−0.0030 (7)	−0.0034 (7)
C10	0.0153 (8)	0.0150 (8)	0.0139 (7)	−0.0056 (6)	−0.0022 (6)	−0.0031 (6)
C11	0.0204 (8)	0.0145 (8)	0.0167 (8)	−0.0056 (6)	−0.0020 (6)	−0.0026 (6)
C12	0.0205 (8)	0.0183 (8)	0.0148 (8)	−0.0075 (7)	−0.0015 (6)	−0.0007 (6)
C13	0.0151 (8)	0.0196 (8)	0.0161 (8)	−0.0071 (7)	−0.0018 (6)	−0.0049 (7)
C14	0.0187 (8)	0.0255 (9)	0.0152 (8)	−0.0078 (7)	0.0001 (6)	−0.0058 (7)
C15	0.0166 (8)	0.0288 (10)	0.0206 (8)	−0.0053 (7)	0.0002 (6)	−0.0118 (7)
C16	0.0171 (8)	0.0205 (9)	0.0246 (9)	−0.0034 (7)	−0.0029 (7)	−0.0089 (7)
C17	0.0177 (8)	0.0168 (8)	0.0203 (8)	−0.0049 (7)	−0.0027 (6)	−0.0049 (7)
C18	0.0151 (8)	0.0178 (8)	0.0150 (8)	−0.0059 (6)	−0.0024 (6)	−0.0041 (6)

### Geometric parameters (Å, º)

**Table:**

Zn—O1	2.0388 (14)	C5—H5B	0.9800
Zn—O2	2.3240 (15)	C5—H5C	0.9800
Zn—O3	1.9397 (13)	C6—C7	1.406 (2)
Zn—N1	2.0570 (15)	C7—C8	1.366 (2)
Zn—N3	2.1460 (14)	C7—H7	0.9500
O1—C1	1.276 (2)	C8—C9	1.494 (2)
O2—C1	1.243 (2)	C9—H9A	0.9800
O3—C3	1.279 (2)	C9—H9B	0.9800
O4—C3	1.239 (2)	C9—H9C	0.9800
N1—C6	1.327 (2)	C10—C11	1.410 (2)
N1—N2	1.3752 (19)	C11—C12	1.366 (3)
N2—C8	1.383 (2)	C11—H11	0.9500
N2—C10	1.414 (2)	C12—C13	1.412 (3)
N3—C10	1.326 (2)	C12—H12	0.9500
N3—C18	1.383 (2)	C13—C18	1.418 (2)
C1—C2	1.507 (3)	C13—C14	1.422 (2)
C2—H2A	0.9800	C14—C15	1.365 (3)
C2—H2B	0.9800	C14—H14	0.9500
C2—H2C	0.9800	C15—C16	1.413 (3)
C3—C4	1.507 (3)	C15—H15	0.9500
C4—H4A	0.9800	C16—C17	1.376 (3)
C4—H4B	0.9800	C16—H16	0.9500
C4—H4C	0.9800	C17—C18	1.411 (2)
C5—C6	1.491 (2)	C17—H17	0.9500
C5—H5A	0.9800		
			
O3—Zn—O1	115.05 (6)	H5A—C5—H5C	109.5
O3—Zn—N1	100.66 (6)	H5B—C5—H5C	109.5
O1—Zn—N1	133.70 (6)	N1—C6—C7	109.92 (15)
O3—Zn—N3	130.20 (6)	N1—C6—C5	121.23 (16)
O1—Zn—N3	99.32 (5)	C7—C6—C5	128.84 (16)
N1—Zn—N3	76.50 (6)	C8—C7—C6	107.15 (16)
O3—Zn—O2	100.51 (6)	C8—C7—H7	126.4
O1—Zn—O2	59.65 (5)	C6—C7—H7	126.4
N1—Zn—O2	86.61 (6)	C7—C8—N2	106.19 (15)
N3—Zn—O2	128.42 (6)	C7—C8—C9	126.73 (16)
C1—O1—Zn	96.11 (11)	N2—C8—C9	127.07 (15)
C1—O2—Zn	83.96 (12)	C8—C9—H9A	109.5
C3—O3—Zn	113.89 (12)	C8—C9—H9B	109.5
C6—N1—N2	106.53 (14)	H9A—C9—H9B	109.5
C6—N1—Zn	137.00 (12)	C8—C9—H9C	109.5
N2—N1—Zn	115.34 (10)	H9A—C9—H9C	109.5
N1—N2—C8	110.21 (13)	H9B—C9—H9C	109.5
N1—N2—C10	116.43 (14)	N3—C10—N2	115.77 (14)
C8—N2—C10	133.36 (14)	N3—C10—C11	123.55 (16)
C10—N3—C18	118.69 (14)	N2—C10—C11	120.68 (15)
C10—N3—Zn	114.76 (11)	C12—C11—C10	118.35 (16)
C18—N3—Zn	126.25 (11)	C12—C11—H11	120.8
O2—C1—O1	120.25 (17)	C10—C11—H11	120.8
O2—C1—C2	120.74 (17)	C11—C12—C13	120.42 (16)
O1—C1—C2	119.00 (17)	C11—C12—H12	119.8
C1—C2—H2A	109.5	C13—C12—H12	119.8
C1—C2—H2B	109.5	C12—C13—C18	117.95 (16)
H2A—C2—H2B	109.5	C12—C13—C14	122.76 (16)
C1—C2—H2C	109.5	C18—C13—C14	119.28 (16)
H2A—C2—H2C	109.5	C15—C14—C13	120.20 (16)
H2B—C2—H2C	109.5	C15—C14—H14	119.9
O4—C3—O3	123.91 (18)	C13—C14—H14	119.9
O4—C3—C4	120.51 (18)	C14—C15—C16	120.16 (17)
O3—C3—C4	115.58 (16)	C14—C15—H15	119.9
C3—C4—H4A	109.5	C16—C15—H15	119.9
C3—C4—H4B	109.5	C17—C16—C15	121.15 (17)
H4A—C4—H4B	109.5	C17—C16—H16	119.4
C3—C4—H4C	109.5	C15—C16—H16	119.4
H4A—C4—H4C	109.5	C16—C17—C18	119.54 (16)
H4B—C4—H4C	109.5	C16—C17—H17	120.2
C6—C5—H5A	109.5	C18—C17—H17	120.2
C6—C5—H5B	109.5	N3—C18—C17	119.36 (15)
H5A—C5—H5B	109.5	N3—C18—C13	121.01 (16)
C6—C5—H5C	109.5	C17—C18—C13	119.63 (16)
			
O3—Zn—O1—C1	86.61 (12)	Zn—N1—C6—C7	−165.93 (13)
N1—Zn—O1—C1	−50.21 (13)	N2—N1—C6—C5	−178.46 (15)
N3—Zn—O1—C1	−130.39 (11)	Zn—N1—C6—C5	15.0 (3)
O2—Zn—O1—C1	−1.07 (10)	N1—C6—C7—C8	−0.2 (2)
O3—Zn—O2—C1	−111.88 (12)	C5—C6—C7—C8	178.77 (18)
O1—Zn—O2—C1	1.10 (10)	C6—C7—C8—N2	−0.27 (19)
N1—Zn—O2—C1	147.89 (12)	C6—C7—C8—C9	178.33 (17)
N3—Zn—O2—C1	78.10 (13)	N1—N2—C8—C7	0.66 (19)
O1—Zn—O3—C3	65.51 (14)	C10—N2—C8—C7	179.74 (17)
N1—Zn—O3—C3	−144.72 (13)	N1—N2—C8—C9	−177.92 (16)
N3—Zn—O3—C3	−63.46 (15)	C10—N2—C8—C9	1.2 (3)
O2—Zn—O3—C3	126.78 (13)	C18—N3—C10—N2	179.97 (14)
O3—Zn—N1—C6	−55.42 (18)	Zn—N3—C10—N2	5.87 (19)
O1—Zn—N1—C6	85.47 (19)	C18—N3—C10—C11	−0.4 (3)
N3—Zn—N1—C6	175.51 (19)	Zn—N3—C10—C11	−174.50 (13)
O2—Zn—N1—C6	44.64 (18)	N1—N2—C10—N3	2.6 (2)
O3—Zn—N1—N2	138.85 (11)	C8—N2—C10—N3	−176.44 (17)
O1—Zn—N1—N2	−80.26 (13)	N1—N2—C10—C11	−177.04 (15)
N3—Zn—N1—N2	9.79 (11)	C8—N2—C10—C11	3.9 (3)
O2—Zn—N1—N2	−121.08 (12)	N3—C10—C11—C12	1.6 (3)
C6—N1—N2—C8	−0.80 (18)	N2—C10—C11—C12	−178.81 (16)
Zn—N1—N2—C8	169.09 (11)	C10—C11—C12—C13	−1.1 (3)
C6—N1—N2—C10	179.95 (14)	C11—C12—C13—C18	−0.4 (3)
Zn—N1—N2—C10	−10.16 (18)	C11—C12—C13—C14	−179.51 (17)
O3—Zn—N3—C10	−101.18 (13)	C12—C13—C14—C15	177.08 (17)
O1—Zn—N3—C10	124.36 (12)	C18—C13—C14—C15	−2.0 (3)
N1—Zn—N3—C10	−8.53 (12)	C13—C14—C15—C16	1.1 (3)
O2—Zn—N3—C10	65.92 (14)	C14—C15—C16—C17	0.7 (3)
O3—Zn—N3—C18	85.24 (15)	C15—C16—C17—C18	−1.5 (3)
O1—Zn—N3—C18	−49.22 (14)	C10—N3—C18—C17	178.31 (15)
N1—Zn—N3—C18	177.89 (15)	Zn—N3—C18—C17	−8.3 (2)
O2—Zn—N3—C18	−107.66 (14)	C10—N3—C18—C13	−1.2 (2)
Zn—O2—C1—O1	−1.76 (17)	Zn—N3—C18—C13	172.15 (12)
Zn—O2—C1—C2	177.01 (16)	C16—C17—C18—N3	−179.02 (16)
Zn—O1—C1—O2	2.01 (19)	C16—C17—C18—C13	0.5 (3)
Zn—O1—C1—C2	−176.78 (14)	C12—C13—C18—N3	1.6 (2)
Zn—O3—C3—O4	5.5 (2)	C14—C13—C18—N3	−179.25 (15)
Zn—O3—C3—C4	−174.94 (12)	C12—C13—C18—C17	−177.93 (16)
N2—N1—C6—C7	0.63 (19)	C14—C13—C18—C17	1.2 (2)

### Hydrogen-bond geometry (Å, º)

**Table:**

*D*—H···*A*	*D*—H	H···*A*	*D*···*A*	*D*—H···*A*
C4—H4*B*···O3^i^	0.98	2.57	3.544 (2)	176
C5—H5*A*···O2^ii^	0.98	2.60	3.417 (3)	141
C7—H7···O2^ii^	0.95	2.56	3.235 (2)	128
C9—H9*C*···O4^iii^	0.98	2.36	3.274 (2)	156
C12—H12···O1^iv^	0.95	2.51	3.310 (2)	142

Symmetry codes: (i) −*x*+2, −*y*+2, −*z*+1; (ii) −*x*+2, −*y*+1, −*z*+1; (iii) −*x*+2, −*y*+1, −*z*+2; (iv) −*x*+1, −*y*+1, −*z*+2.
